# Supplementary figures and images for: MicroRNA-154-5p suppresses cervical carcinoma growth and metastasis by silencing Cullin2 in vitro and in vivo
Source: PeerJ. 2023 Jun 27;11:e15641. doi: 10.7717/peerj.15641 (PMC10312157; doi:10.7717/peerj.15641)

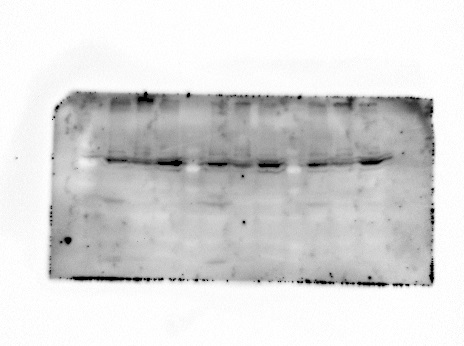

Supplement: Supplemental Information 4 [file peerj-11-15641-s004.zip › Uncropped Blots/Figure.6E_separate blot/CUL2 for Figure.6E.jpg]

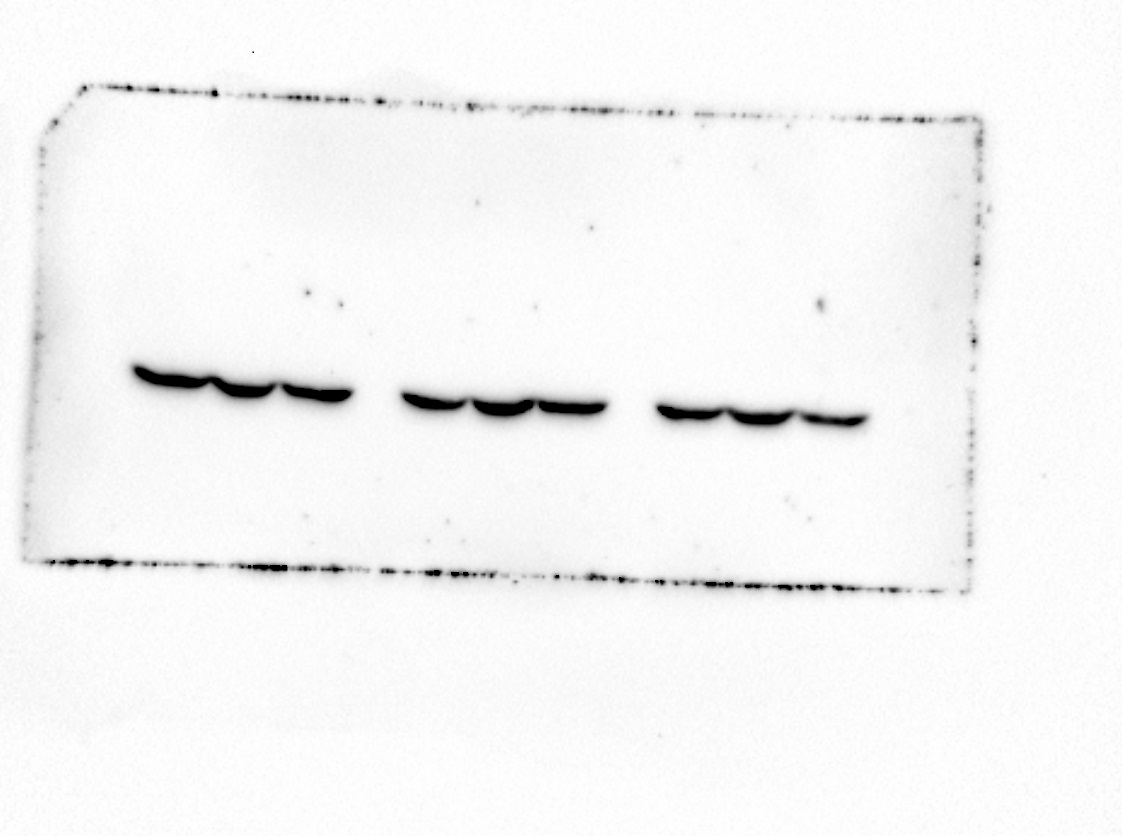

Supplement: Supplemental Information 4 [file peerj-11-15641-s004.zip › Uncropped Blots/Figure.6E_separate blot/a┬-actin for Figure.6E.jpg]

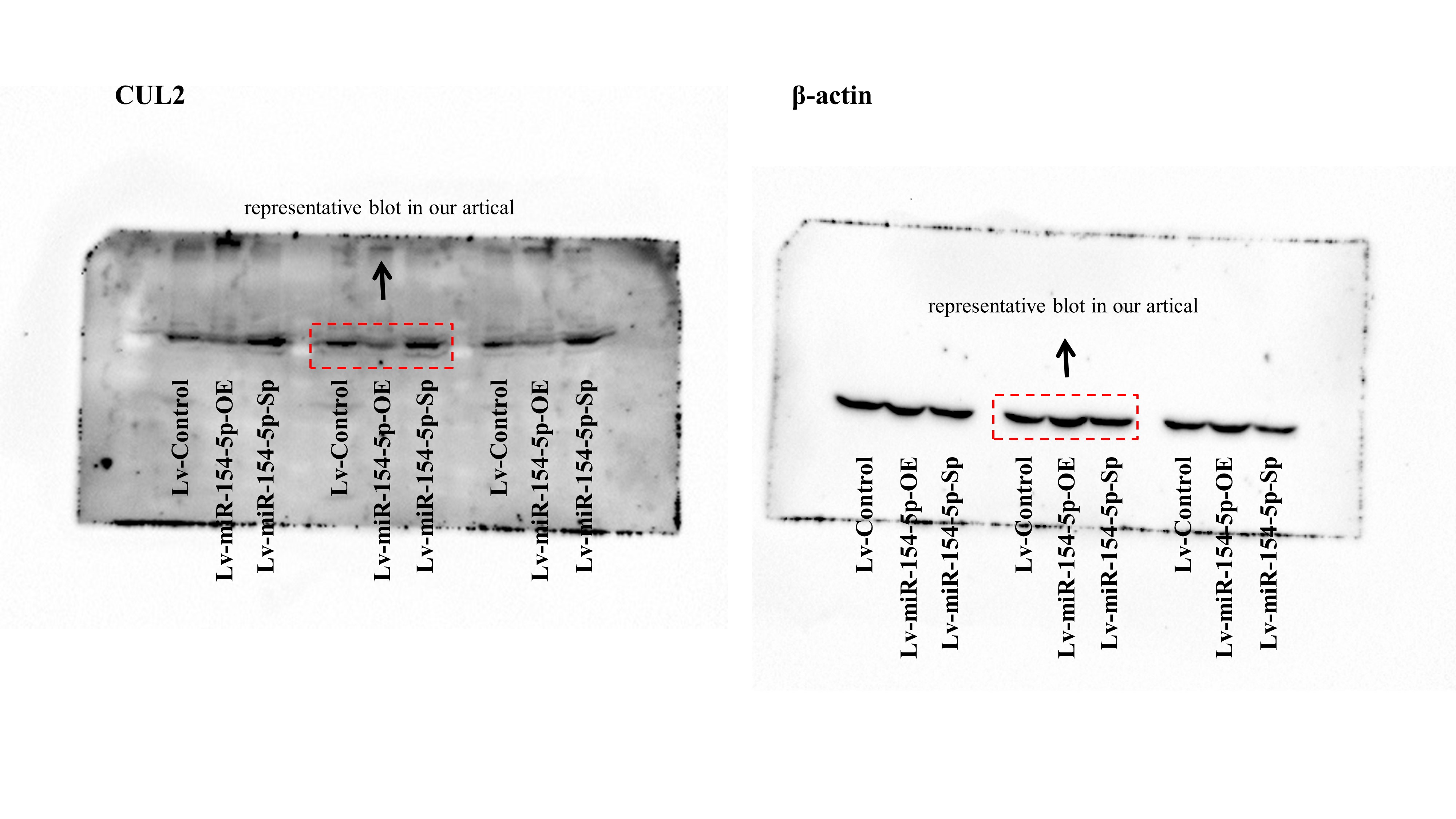

Supplement: Supplemental Information 4 [file peerj-11-15641-s004.zip › Uncropped Blots/Figure.6E-uncropped blots.jpg]

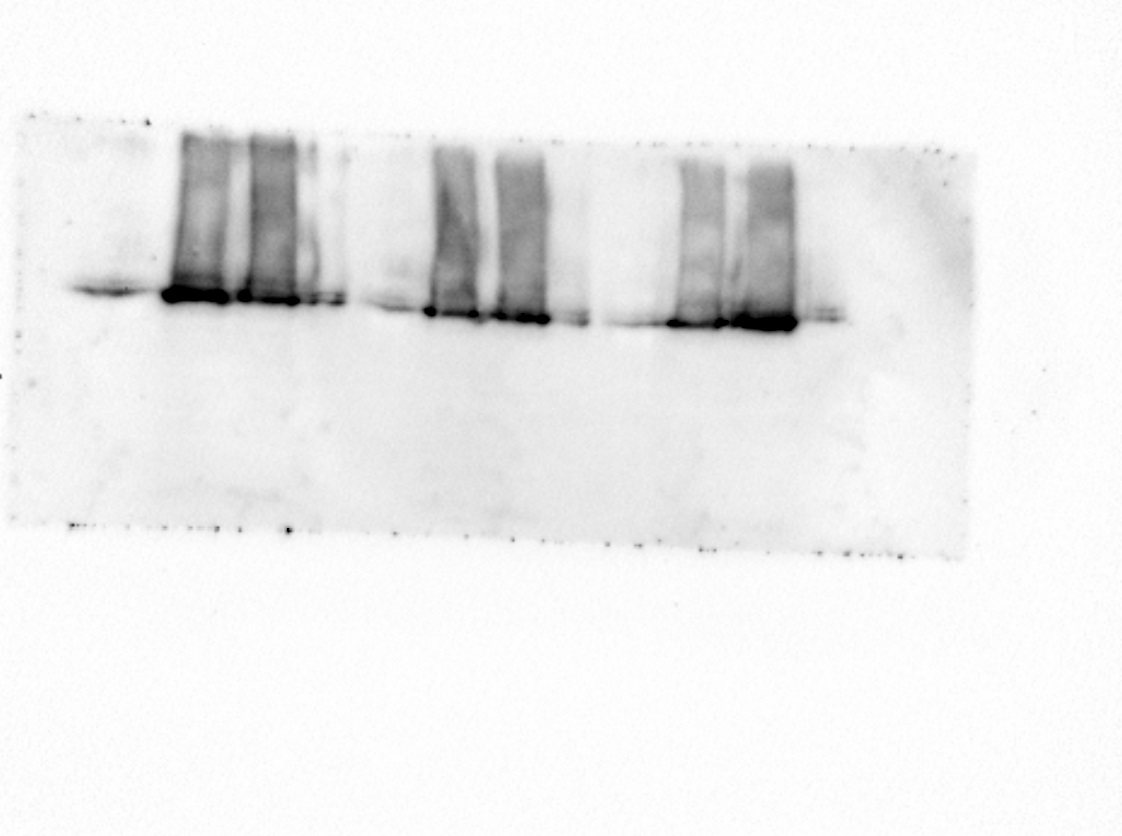

Supplement: Supplemental Information 4 [file peerj-11-15641-s004.zip › Uncropped Blots/Figure.8C_separate blot/CUL2 for Figure.8C.jpg]

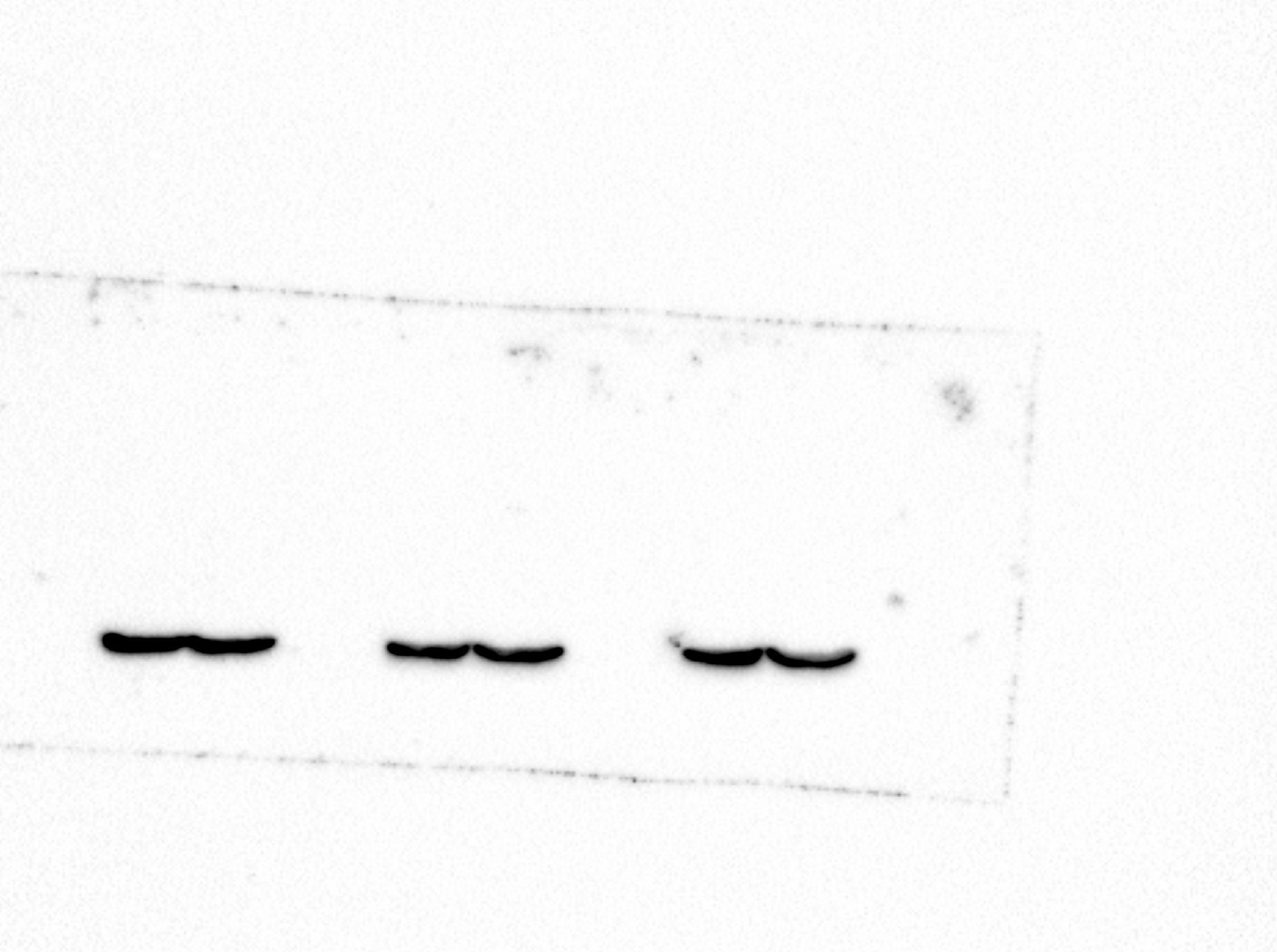

Supplement: Supplemental Information 4 [file peerj-11-15641-s004.zip › Uncropped Blots/Figure.8C_separate blot/a┬-actin for Figure.8C.jpg]

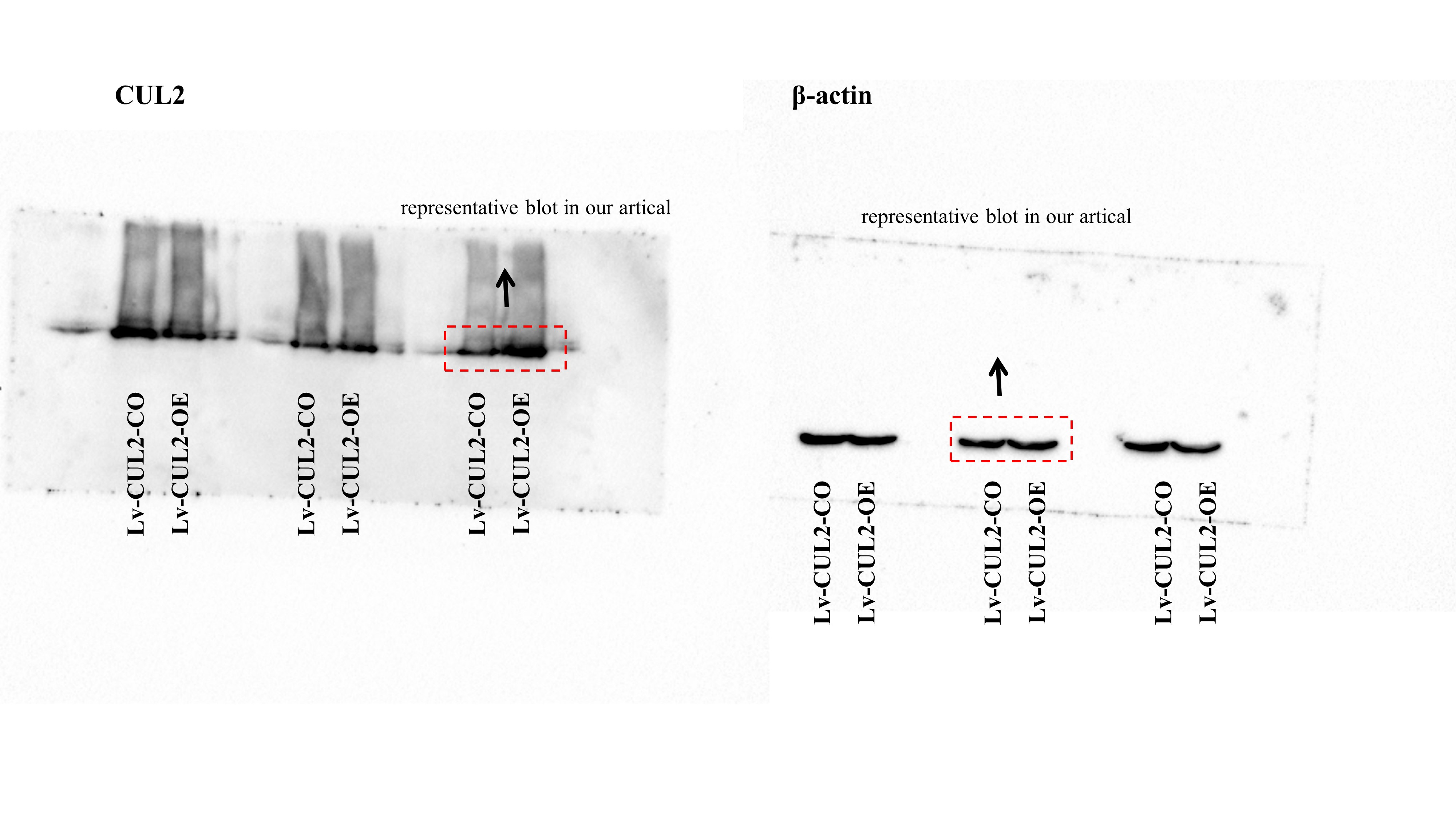

Supplement: Supplemental Information 4 [file peerj-11-15641-s004.zip › Uncropped Blots/Figure.8C-uncropped blots.jpg]

Figure.6E_ raw western blot


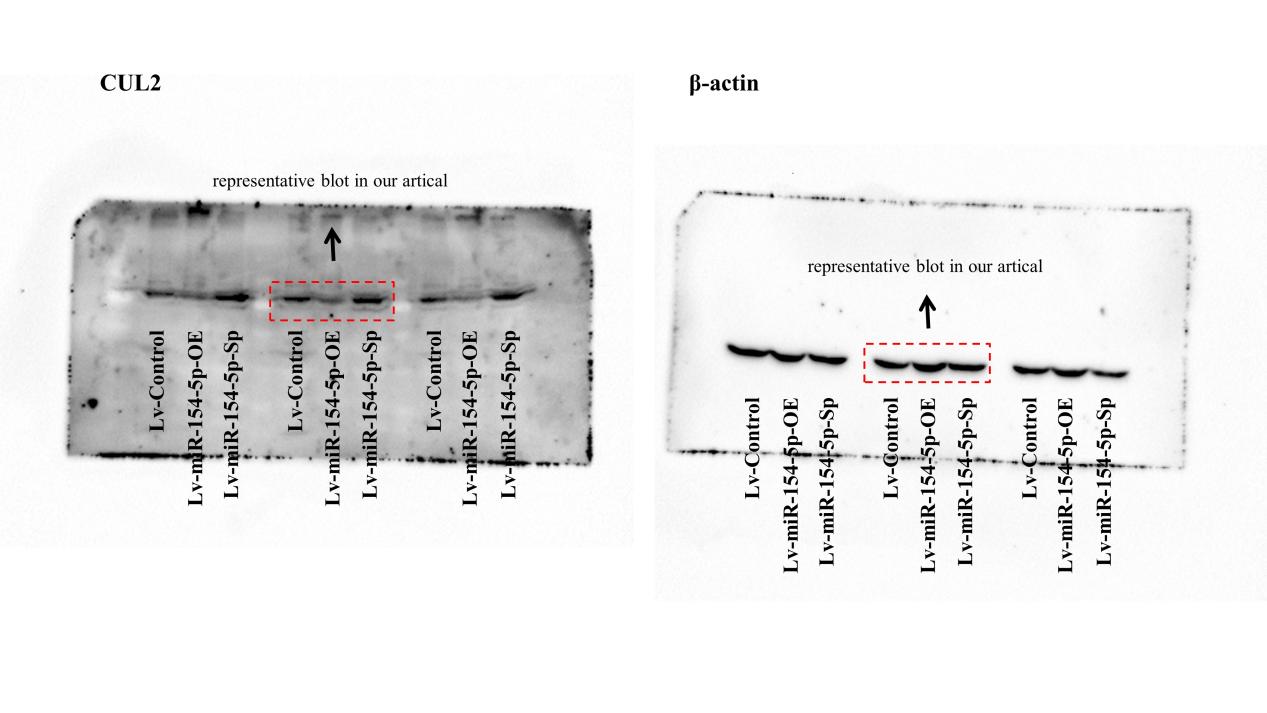


Figure.8C_ raw western blot


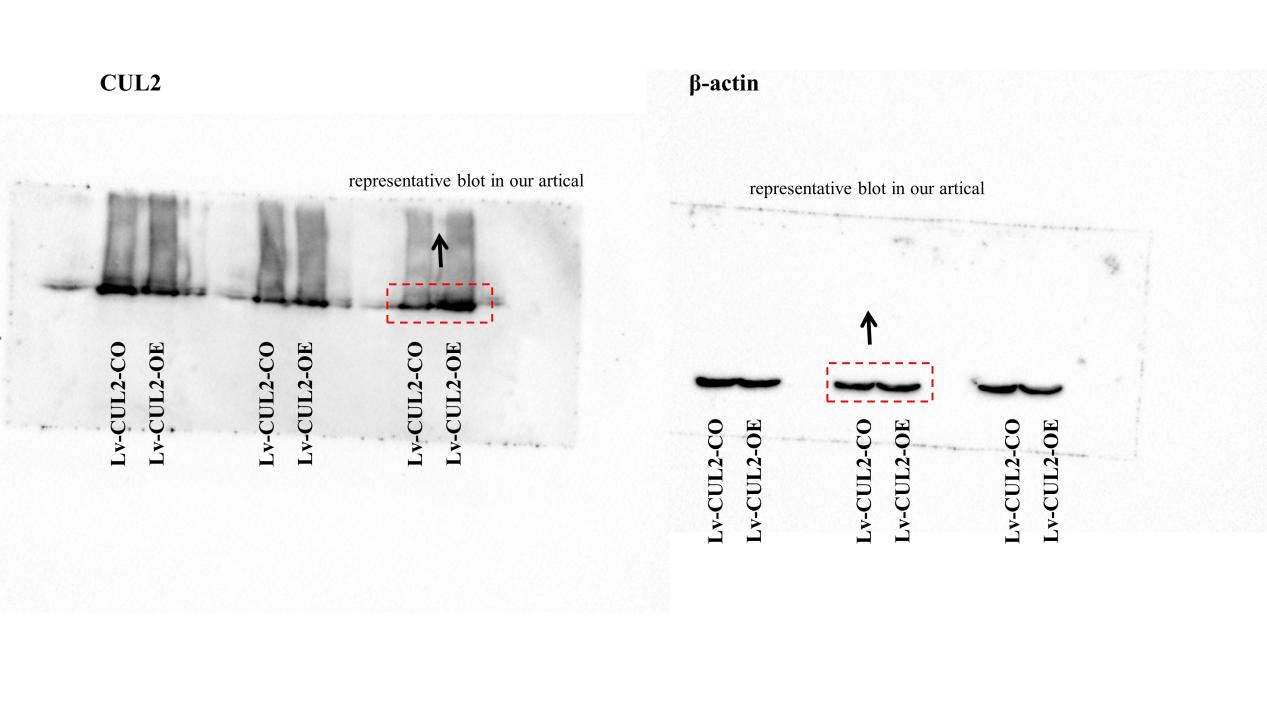

Supplement: Supplemental Information 4 [file peerj-11-15641-s004.zip › Uncropped Blots/Uncropped Blots.docx]
